# The promise of endogenous and exogenous riboflavin in anti-infection

**DOI:** 10.1080/21505594.2021.1963909

**Published:** 2021-09-07

**Authors:** Junwen Lei, Caiyan Xin, Wei Xiao, Wenbi Chen, Zhangyong Song

**Affiliations:** Molecular Biotechnology Platform, Public Center of Experimental Technology, School of Basic Medical Sciences, Southwest Medical University, Luzhou People’s Republic of China

**Keywords:** Antibacterial agent, antifungal agent fmn riboswitch, immune response, mait cell, riboflavin

## Abstract

To resolve the growing problem of drug resistance in the treatment of bacterial and fungal pathogens, specific cellular targets and pathways can be used as targets for new antimicrobial agents. Endogenous riboflavin biosynthesis is a conserved pathway that exists in most bacteria and fungi. In this review, the roles of endogenous and exogenous riboflavin in infectious disease as well as several antibacterial agents, which act as analogues of the riboflavin biosynthesis pathway, are summarized. In addition, the effects of exogenous riboflavin on immune cells, cytokines, and heat shock proteins are described. Moreover, the immune response of endogenous riboflavin metabolites in infectious diseases, recognized by MHC-related protein-1, and then presented to mucosal associated invariant T cells, is highlighted. This information will provide a strategy to identify novel drug targets as well as highlight the possible clinical use of riboflavin.

## Introduction

Drug resistance of bacteria, fungi and even viruses is increasingly becoming a serious problem worldwide. Antimicrobial-resistant bacteria (such as *Staphylococcus aureus*, methicillin-resistant *S. aureus* (MRSA), *Streptococcus pneumoniae* and *Salmonella typhimurium*) have been found in hospitals and community settings [1,2]. Excessive use of antifungal drugs to treat humans and in other applications has led to increasing resistance [3]. The resistance of *Candida* and *Aspergillus* species notably presents a major problem in clinical treatment [4]. In addition, the issue of virus resistance cannot be ignored; the resistance of viruses (such as hepatitis B virus [5] and influenza virus [6]) has become a major challenge to human public health. Meanwhile, the emergence of many new resistance mechanisms also increases the difficulty in treating resistant microorganisms and viruses [7]. Our current, limited antimicrobial drugs can no longer solve the growing problem of drug resistance [8–10]. Therefore, it is urgent to develop additional antimicrobial drugs. Sulfonamides, the first generation of antibiotics [11], have recently been shown to target *de novo* folate synthesis in pathogens [12,13]. Therefore, microbial cellular targets and pathways, such as endogenous riboflavin biosynthesis, may provide targets for novel drug discovery [14,15].

Riboflavin (vitamin B_2_) was isolated from milk whey in the late 1870s as a water-soluble compound. It is indispensable to adenine dinucleotide (FAD) and flavin mononucleotide (FMN), which participate in electron transport, and metabolism of lipids, drugs, and xenobiotics. Because of the absence of a system to produce riboflavin, riboflavin in humans is mainly obtained from the diet. However, bacteria and fungi, such as *Aspergillus fumigatus, Candia albicans, Escherichia coli, Mycobacterium tuberculosis*, and *S. typhimurium*, have the ability to produce riboflavin [16–18]. Silencing or downregulation of genes of the endogenous riboflavin biosynthetic pathway may be beneficial tin treating fungal skin infections [19]. Furthermore, exogenous riboflavin has also been shown to exhibit anti-infectious effects in infectious diseases [20,21].

In this review, the role of the endogenous riboflavin synthesis pathway, which may be a target for the development of antimicrobial agents, and exogenous riboflavin, against infectious diseases is provided. The effects of exogenous riboflavin on immune cell and cytokine gene and heat shock protein gene expression are also presented. Moreover, the relationship between endogenous riboflavin and mucosal associated invariant T (MAIT) cell is highlighted. These data provide a comprehensive review of the use of endogenous and exogenous riboflavin in anti-infection.

## Endogenous riboflavin synthesis pathways and the FMN riboswitch target in pathogens

### Biosynthesis and transport of riboflavin

**Figure 1.**
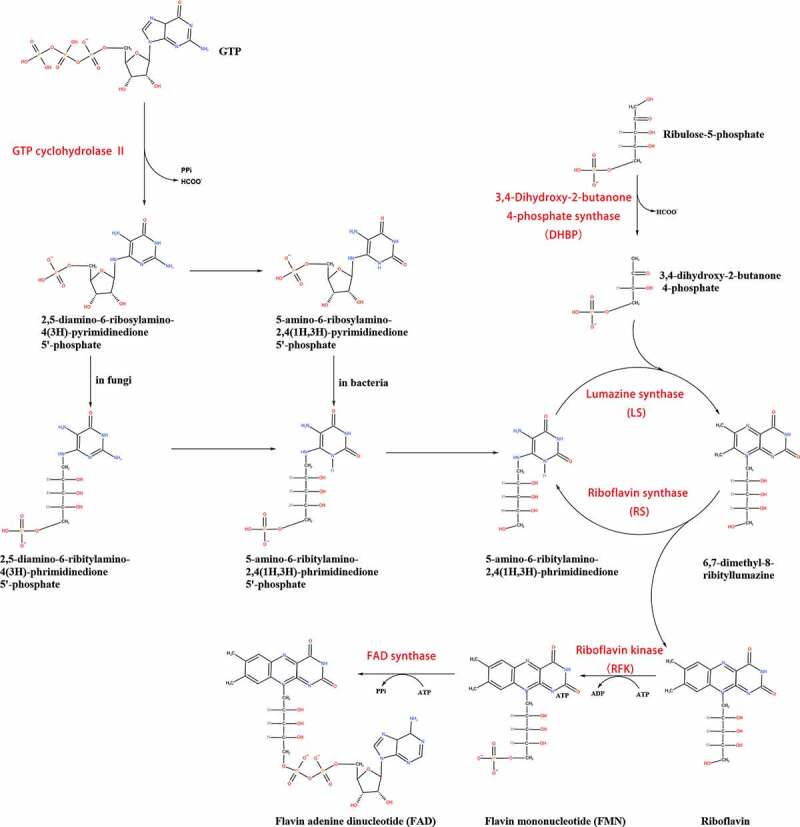
Biosynthesis of riboflavin, FMN and FAD

The riboflavin biosynthesis pathway is summarized in Figure 1. Riboflavin is formed by consuming guanosine triphosphate (GTP) and ribulose 5-phosphate (Ru5P) [9]. GTP cyclohydrolase II catalyzes GTP to form 2,5-diamino-6-ribosylamino-4(3 *H*)-pyrimidinedione phosphate **(1)**. The first step is common to both bacteria and fungi, whereas the second steps are different. In bacteria, **(1)** is converted into 5-amino-6-ribosylamino-2,4(1 *H*,3 *H*)-pyrimidinedione 5ʹ-phosphate **(2)** to form 5-amino-6-ribitylamino-2,4(1 *H*,3 *H*)-pyrimidinedione 5ʹ-phosphate **(4)**. However, in fungi, **(1)** is converted into 2,5-diamino-6-ribosylamino-4(3 *H*)-pyrimidinedione 5ʹ-phosphate **(3)** [22,23]. Then, **(4)** is dephosphorylated to form 5-amino-6-ribitylanimo-2,4(1 *H*,3 *H*)-pyrimidinedione **(5)**. Another compound involved in riboflavin synthesis is 3,4-dihydroxy-2-butanone 4-phosphate **(6)**, which is converted from Ru5P catalyzed by 3,4-dihydroxy-2-butanone 4-phosphate synthase. **(5)** and **(6)** are catalyzed by Lumazine synthase (LS) to form 6,7-dimethyl-8-ribityllumazine **(7)**. Then, riboflavin synthase (RS) catalyzes formation of the LS substrate **(5)** and riboflavin. Riboflavin is usually converted to FMN and FAD. Riboflavin kinase catalyzes riboflavin to form FMN, which is then converted into FAD by FAD synthase [24,25]. Until now, the endogenous riboflavin synthesis pathway in viruses has not been found.

Riboflavin is formed by GTP and Ru5P, and key enzymes are in red (reproduced from reference [37]). 5-OP-RU: 5-(2-oxopropylideneamino)-6-D-ribitylaminouracil, 5-OE-RU: 5-(2-oxoethylideneamino)-6-D-ribitylaminouracil

In addition, some microorganisms are able to take up exogenous riboflavin. In early 1979, components of the cell membrane were found to be involved in riboflavin transport [26]. Riboflavin transporters introduce riboflavin into cells; these transporters include RibU of *Lactococcus lactis*, RibM of *Actinobacteria and Streptomyces davaonensis* [27], ImpX of *Fusobacterium nucleatum* [28], RibZ of *Clostridium difficile*, RibV of *Mesoplasma florum*, and RibXY of *Chloroflexus aurantiacus* [29]. However, exogenous riboflavin may suppress the biosynthesis of riboflavin in some cases [30]. The relationship between the uptake system and the *de novo* biosynthesis pathway remains unclear [29]. Most fungi are able to biosynthesize riboflavin, but the uptake of riboflavin has been relatively less studied. Wild type *Saccharomyces cerevisiae* has a riboflavin efflux system but cannot take up exogenous riboflavin [31]. The expression of *MCH5* appears to be necessary for the uptake of riboflavin in auxotrophic *S. cerevisiae* mutants [32]. Unlike bacteria, fungi transport riboflavin through a passive process that dose not consume any energy [26,32]. The riboflavin transport systems in pathogenic fungi and viruses require further study. Human riboflavin transporters (*hRFVT1, hRFVT2*, and *hRFVT3*) belong to the SLC52 family of solute carriers, which show low homology with bacterial or fungal riboflavin transporters [33]. Therefore, the riboflavin transport in bacteria or fungi may provide ideas for developing antimicrobial agents.

### Targeting the FMN riboswitch

FMN riboswitches are broadly conserved and consist of non-coding RNA structural elements. In the presence of FMN, they regulate the biosynthesis and transport of riboflavin in bacteria [34,35]. Importantly, they are specific to bacteria and absent in humans [36]. Here, several antibacterial agents, which include analogues of the riboflavin biosynthesis pathway, such as ribocil, roseoflavin (RoF), 8-demethyl-8-aminoriboflavin (AF), and 5-(3-(4-fluorophenyl)butyl)-7,8-dimethylpyrido[3,4-b]quinoxaline-1,3(2 H,5 H)-dione (5FDQD), protect against the pathogens by targeting the FMN riboswitch.

Ribocil directly binds the FMN riboswitch and inhibits *ribB* expression, inducing bacterial death by blocking the riboflavin biosynthesis pathway in *S. aureus* and *E. coli* [37]. Further resistance mutation and whole-genome sequencing identified the ribocil target at the FMN riboswitch in *E. coli* [38,39]. However, exogenous riboflavin reduced the antibacterial activity of ribocil *in vitro* [37]. Interestingly and contrastingly, ribocil and RoF not only block riboflavin biosynthesis but also inhibit uptake from the environment in *S. aureus* and MRSA [34,40].

RoF is produced by *S. davaonensis* and *S. cinnabarinus* [41], and its main antibacterial spectrum is Gram-positive bacteria. The FMN riboswitch is a target for RoF in *Bacillus subtilis* [42] *E. coli* [15], *F. nucleatum* [43], and *Listeria monocytogenes* [44,45], which may be primarily responsible for its antibiotic activity. On the one hand, promiscuous riboflavin kinases catalyze RoF to form toxic RoF mononucleotide (RoFMN), which negatively regulates the *ribB* FMN riboswitch in *E. coli* [15,46]. *L. monocytogenes* does not contain genes that encode riboflavin biosynthetic enzymes. RoF was also shown to target the FMN riboswitch and inhibit the growth of *L. monocytogenes*. Surprisingly, RoF can increase the pathogenicity of *L. monocytogenes* in the absence of the FMN riboswitch [44,45]. However, the mechanisms require further study. On the other hand, the toxic RoFMN and RoF adenine dinucleotide (RoFAD) restricted growth of *B. subtilis* and *S. davaonensis* [45,47–49]. RoFMN and RoFAD can also be isolated from *E. coli* flavoproteins. Thus, flavoproteins may be another target for RoF in addition to FMN riboswitches [50]. However, human FAD synthase can accept RoFMN and disrupt the activity of flavoenzymes, which may be disadvantageous to human metabolism [40,51].

AF, also produced by *S. davaonensis*, is less toxic to host. Similar to RoF, AF negatively regulates *ribB* expression, which is controlled by the *ribB* FMN riboswitch. Human flavokinase converts AF to 8-demethyl-8-amino-riboflavin mononucleotide (AFMN). In contrast to RoF, human FAD synthase cannot accept AFMN. Therefore, it may provide a better structure to develop antibacterial compounds [51]. Furthermore, 5FDQD defends against *Clostridium difficile* infection through binding to FMN and triggering the function of the FMN riboswitch. However, many aerobes and Gram-negative anaerobes are not sensitive to 5FDQD [52]. This is likely because the FMN riboswitch is not highly conserved across different bacteria. Thus, antibiotics targeting the FMN riboswitch are a narrow spectrum. Overall, FMN riboswitches are the main target of several existing inhibitors of the riboflavin synthesis pathway. However, the function and application of FMN riboswitches in fungi have been rarely studied. The question is whether the FMN riboswitch exists in fungi? If it does exist, the function of the FMN riboswitch in regulation of riboflavin biosynthesis will require further investigation in fungi.

### Inhibiting enzymes in the riboflavin synthesis pathway

LS and RS were identified in fungi and bacteria [30] and can be inhibited by antimetabolites [53]. Mutations of LS genes have emphasized the essential role of riboflavin biosynthesis in pathogen survival. For example, there are two LS isoenzyme-related genes (*ribH1* and *ribH2*) in *Brucella abortus*. The double mutant of *ribH1* and *ribH2* does not survive without exogenous riboflavin [54]. Thus, inhibitors of these enzymes are logical candidates for development as antibiotics [55]. Crystal structure analysis of icosahedral LS from *S. typhimurium* also supports the above-mentioned conclusion [56]. Several potent LS inhibitors were discovered by a high throughput screening approach. However, cell membrane permeability remains an issue for the therapeutic use of these compounds [57]. Further investigations are needed to enhance cell membrane permeability *in vivo* and *in vitro*. RS is highly conserved across pathogenic microorganisms, and may be a suitable target for alternative antibiotics [58]. High-throughput screening technology was used to discover and develop covalent hydrates of trifluoromethylated pyrazoles, which can inhibit RS in *M. tuberculosis* [59]. Using the same technology, several inhibitors of *B. abortus* RS were discovered [60]. One compound could inhibit LS in *B. subtilis* and *M. tuberculosis* and RS in *E. coli* [61]. Mutation of both LS and RS is a rare event, but can lead to a low rate of resistance in strains [16]. Therefore, this compound can be used as a lead structure for designing dual inhibitors.

3,4-Dihydroxy-2-butanone-4-phosphate synthase (DHBP) catalyzes Ru5P to form **(6)** and formate (Figure 1). A competitive inhibitor of DHBP in *Vibrio cholerae*, 4-phospho-d-erythronohydroxamic acid, has been described [62,63]. DHBP is essential to *Helicobacter, Mycobacterium, Salmonella* species, and *S. pneumoniae* [[64-]]. Moreover, in eukaryotes and prokaryotes, FMN and FAD are catalyzed by FAD synthase and RFK, respectively (Figure 1). The two are involved in a plethora of vital processes. Therefore, the most important thing is to identify the different characteristics of FAD synthase/RFK in pathogens and hosts. However, the characteristics of FAD synthase in *S. pneumoniae* are different from those in other bacteria [65]. In addition, several FAD synthase inhibitors were shown to inhibit the growth of *M. tuberculosis* and *S. pneumoniae* [66].

To date, studies of endogenous riboflavin synthesis pathway inhibitors have been primarily performed in bacteria, and have resulted in the design of several inhibitors. Riboflavin biosynthesis and uptake are essential not only for invasion but also during dissemination. *RIBB* and *RIB1* encode the GTP cyclohydrolase II enzyme, which converts GTP into **(1)** (Figure 1). Studies have shown that the *RIBB* mutant attenuates the virulence of *A. fumigatus* and inhibits its survival in hosts [67]. *CaRIB1Δ/Δ* deletion strains show no toxicity in HeLa cells and are completely avirulent in a mouse model [68–70]. *RIB2* encodes the 2,5-diamino-6-ribitylamino-4(3 H)-pyrimidinone 5ʹ-phosphate deaminase of *Histoplasma capsulatum*, which catalyzes **(1)** into **(3)** (Figure 1). Disruption of the *RIB2* gene prevents growth and proliferation of *H. capsulatum* in macrophages and severely attenuates its virulence [71]. FlcA, FlcB, and FlcC are important for FAD accumulation and *A. fumigatus* virulence, and FlcA-C belong to the flavin transporter family. The virulence of the ∆*flcA*, ∆*flcB*, and ∆*flcC* mutant strain is lower, than that of the wild type, thus resulting in a higher survival rate of infected mice [71]. In conclusion, GTP cyclohydrolase II enzyme, DHBP, LS, RFK, RS, and FAD synthase, which are involved in the riboflavin synthesis pathway, can be used as antibacterial and antifungal drug targets (Table 1). However, RFK and FAD synthase are also present in humans [30]. Thus, the application of RFK and FAD synthase inhibitor against bacterial and fungal infections in the human body should be considered carefully.

**Table 1.** Inhibitors of enzymes in the riboflavin synthesis pathway

Inhibitors	Target species	Reference
DHBP inhibitors	*Vibrio cholerae*	[62]
LS inhibitors	*Schizosaccharomyces pombe, C. albicans, B. abortus; and M. tuberculosis*	[55,57,61]
RS inhibitors	*M. tuberculosis, B. abortus* and *E. coli*	[59–61]
RFK inhibitors	*S. pneumoniae and Corynebacterium ammoniagenes*	[65]
FAD inhibitors	*C. ammoniagenes, M. tuberculosis* and *S. pneumoniae*	[66]

## Immune response of endogenous riboflavin

**Figure 2.**
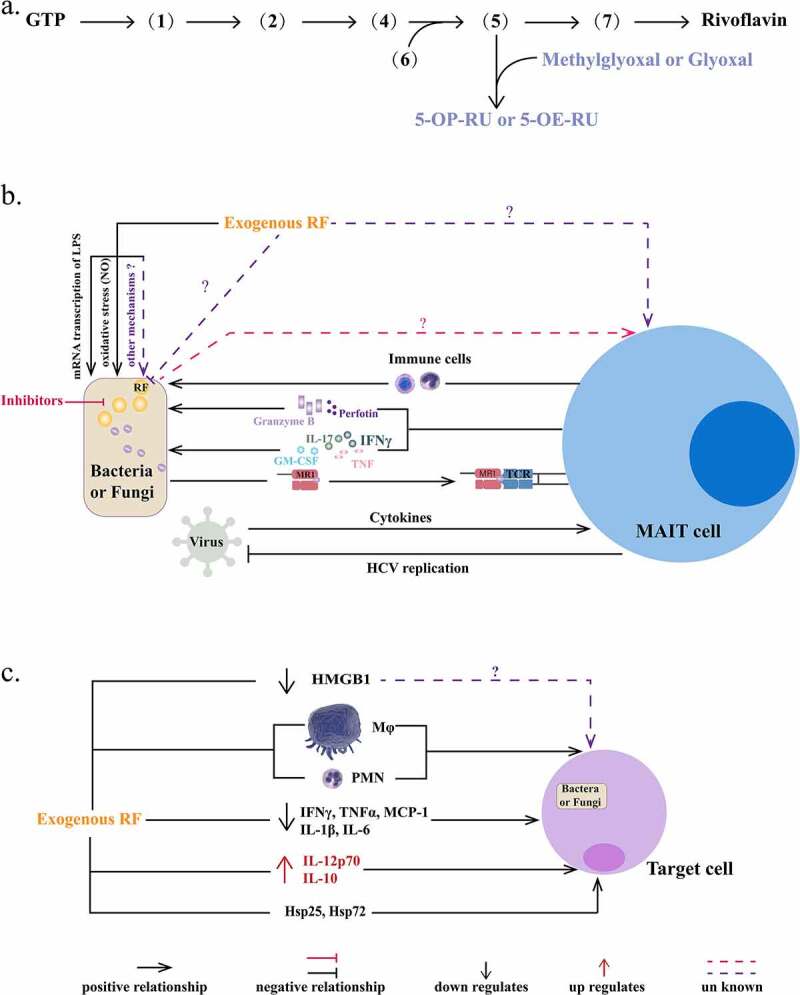
**Immune responses of endogenous/exogenous riboflavin and exogenous riboflavin against infection. a. The formation of pyrimidine adducts**. 5-(2-oxoethylideneamino)-6-D-ribitylaminouracil (5-OE-RU) and 5-(2-oxopropylideneamino)-6-D-ribitylaminouracil (5-OP-RU) are formed by **(5)** combining with glyoxal or methylglyoxal in bacteria. RF: riboflavin. **b. Interaction between riboflavin and MAIT cells**. MR1 recognizes and presents the signals of the riboflavin synthesis pathway to MAIT cells triggering different types of immune responses in bacteria and fungi. 1) MIAT cells produce granzyme B and perforin to kill the target cell directly. 2) MAIT cells release cytokines, including IFN-γ, GM-CSF, IL-17, and TNF. 3) MAIT cells facilitate other immune cells to protect the host from infection. However, fungi are recognized in an MR1-dependent manner and, therefore, MAIT cells response to fungi are not exactly same as to bacteria. Viruses cannot biosynthesize riboflavin, and depend on cytokines to activate MAIT cells. In most virus infections, MAIT cells levels were reduced and their functions were impaired, such as antimicrobial activity. In addition, activated MAIT cells can limit HCV replication, and the mechanism should be further explored. **c. The immune responses of exogenous riboflavin against infection**. Exogenous riboflavin affects infectious disease by regulating the function of immune cells and the release of cytokines/inflammatory factors. HMGB1: high mobility group box 1 protein, Mø: macrophage, PMN: polymorphonuclear cell. INF-γ: interferon-γ, TNF-α: tumor necrosis factor-α, MCP-1: monocyte chemoattractant protein-1, IL: interleukin, Hsp: heat shock protein

It is necessary to understand how the endogenous riboflavin biosynthesis pathway of pathogens affects the human immune response. 5-(2-oxopropylideneamino)-6-D-ribitylaminouracil and 5-(2-oxoethylideneamino)-6-D-ribitylaminouracil are intermediates in the microbial riboflavin biosynthesis pathway, and are considered to be the biological signature of microbial infection in mammals, which are formed by (5) combined with methylglyoxal or glyoxal [72] (Figure 2a). The major histocompatibility class I-like antigen-presenting molecule, MHC-related protein-1 (MR1), captures these pyrimidine intermediates and forms a complex. MAIT cell antigen receptors accept MR1-antigen complexes and then trigger MAIT cell immune responses, which lead to protection of the host from pathogens at mucosal surfaces [73–75]. In the early life of humans, the development of MAIT cells requires exposure to microorganisms with the ability to synthesize riboflavin. Subsequently, MAIT respond to cutaneous microbes, which are beneficial to tissue repair. Furthermore, MR1, which presents riboflavin metabolites, is necessary for MAIT cells to recognize pathogens [76]. Besides the activation and enrichment of MAIT cells, the production of cytokines is also a response to microbial antigens [77]. For example, *Legionella longbeachae* induces MAIT cell activation and rapid pulmonary accumulation in an MR1-dependent manner in pulmonary *L. longbeachae*-infected mice. Interferon (IFN)-γ, granulocyte-macrophage colony stimulating factor (GM-CSF), tumor necrosis factor (TNF), and Interleukin (IL)-17 produced by activated MAIT cells enhance host immune protection [78]. Granzyme B and perforin, the secretions of MAIT cells, can also kill target cells to fight infection [79,80]. In addition, they promote the accumulation of CCR1^+^ and CCR5^+^ immune cells to the lung, preventing *mycobacterial* infection [80]. It is worth noting that MAIT cells are polarized to the Th1 phenotype and migrate to the infectious site during *M. tuberculosis* infection [81,82]. In addition, fungi (including *Aspergillus ssp., C. albicans*, and *Mucorales* species) can be recognized by MAIT cells in an MR1-dependent manner [83–85].

However, immune responses of MAIT cells are different in different kinds of infections. For example, *A. fumigatus* infection induces MAIT cells to produce a large amount of TNF and less IFN-γ, in contrast to *C. albicans* infection, which induces MAIT cells to mainly produce IFN-γ. Differences in the MAIT cell response to *C. albicans* and *E. coli* can be seen in the sensitivity of recognition, release of cytokines, and immune response [84,86]. Viruses cannot produce riboflavin metabolites to form MR1-antigen complexes. MAIT cells depend on cytokines, but not MR1, which play a protective role in influenza virus, dengue virus, and hepatitis C virus infection [87–89] (Figure 2b). Activated MAIT cells can limit hepatitis C replication, and the mechanism should be further explored [88]. However, MAIT cells are activated and exhausted in most viral infections [90,91]. The antivirus activity of MAIT cells was induced in human immunodeficiency virus infection [92,93]. Thus, MAIT cells can be activated by different types of microbial stimuli and against bacteria, fungi, and viruses in different response patterns [86]. Thus, MAIT cells also promote cytokine and chemokine release, as well as promote migration of immune cells to sites of infection, which enhances host immunity and exerts an anti-infectious effect (Figure 2b). However, MAIT cells cannot be activated by *A. fumigatus* strains lacking *ribB* [84]. Hence, it is worth considering that whether inhibitors of the riboflavin biosynthesis pathway affect the formation of MR1-antigen compounds, resulting in pathogen evasion from MAIT cells. Further investigations are needed.

## Anti-infection effect of exogenous riboflavin

### Direct anti-infective effect of riboflavin

Riboflavin can be used as an antibiotic in the treatment of various infectious diseases. Intravenous injection of highly purified riboflavin (80 mg/kg) greatly reduced the virulence of *E. coli* and *S. aureus* as well as the production of proinflammatory cytokines and nitric oxide (NO) induced by lipopolysaccharide (LPS) [20]. Exogenous riboflavin (10 mg/kg) could also protect mice against LPS-induced shock by increasing the level of heat shock protein 25 (Hsp25) to decrease mortality [94]. In addition, exogenous riboflavin (10 mg/kg) and riboflavin combined with aminolevane increased survival of mice with LPS-induced shock [95]. Riboflavin (20 mg/kg) also decreased IL-6 and macrophage inflammatory protein-2 concentrations and mRNA transcription levels in mice injected with LPS. It also reduced plasma elevated NO levels by reducing expression of inducible NO synthase (*iNOS*) gene [96]. Supplementation with 300 mM riboflavin reduced the mortality of LPS-stimulated macrophages and induced expression of Hsp72 in macrophages [97]. These data illustrate that intravenous injection of riboflavin has antibacterial effects.

*In vivo*, the dominant immune responses of C57BL/6 J, BALB/c, and CBA mice are different, which results in the different effects of riboflavin supplementation on fungal peritonitis. In general, riboflavin affects matrix metalloproteinase-9 activity, *iNOS* gene expression, and the migration of polymorphonuclear cells (PMNs) and macrophages [98]. The effect of riboflavin on zymosan-induced peritonitis in Swiss mice has also been studied. The results suggested that the effects of riboflavin on proinflammatory and anti-inflammatory cytokines were most significant. Different from LPS-induced infection, 20 mg/kg riboflavin was usually ineffective in zymosan-induced infection in Swiss mice [21], while 300 nM riboflavin supplementation of macrophages stimulated by zymosan significantly decreased Toll-like receptor 6, NO, iNOS, IL-1β, monocyte chemoattractant protein-1 (MCP-1), and keratinocyte chemoattractant levels [97]. Riboflavin supplementation reduced the release and expression of high mobility group box 1 protein (HMGB1) in zymosan-induced peritonitis mice and *in vitro* macrophage model. HMGB1 is responsible for activation of neutrophils or macrophages in recent studies [99]. In-depth studies of the mechanisms of action will be necessary in the future.

Exogenous riboflavin directly controls infection in three ways: 1) it inhibits transcription of bacterial LPS (Figure 2b); 2) it reduces the level of *NO* or expression *of iNOS*; and 3) it regulates the functions of innate immune cells (such as macrophages and neutrophils) and the levels of immunoreactive materials, including TNF, ILs, and IFN. The key factors affecting the anti-bacterial or anti-fungal effects of riboflavin are the time of administration and the dose [20,21,94,95]. Exogenous riboflavin regulation of immune responses is highlighted in Figure 2c. These immune responses also have a vital role in virus infection [100]. Thus, riboflavin may affect the occurrence and development of viral infection. In addition, the function and activation of MAIT cells participate in host defense during microbial or viral infection [88–91]. Therefore, exogenous riboflavin may directly affect the function of MAIT cells or influence MAIT cells’ recognition of pathogens. Further experiments are needed to confirm these conclusions.

### Indirect anti-infective effects of riboflavin

Riboflavin can also be used as a synergist to enhance the activity of antimicrobial or antiviral drugs. In 1996, Adelavin, a compound that contains liver extract and FAD, was used in patients with chronic hepatic-C and potentially enhanced the anti-viral effect of IFN [100]. Riboflavin showed significant synergistic activity with linezolid against MRSA infection [101]. Riboflavin in combination with azithromycin treatment eliminated *S. aureus* from blood, spleen, and synovial tissue of infected mice, reduced serum levels of TNF-α, INF-γ, and IL-6, and increased serum anti-inflammatory cytokines IL-12p70 and IL-10 [102].

Riboflavin, a natural photosensitizer, is widely used in photodynamic inactivation of microorganisms. Photo-illuminated riboflavin inhibits the biofilm formation of *S. aureus* and *E. coli* by inducing reactive oxygen species accumulation and oxidative stress, and destroying the respiratory system [103,104]. In combination with ultraviolet (UV) light A, riboflavin damages nucleic acids of pathogens (such as *S. aureus*, MRSA, and *P. aeruginosa* [105]) through the same mechanism [106,107]. Low concentrations of riboflavin (0.03%–0.07%) can improve the bactericidal effects of UV light A [108]. Interestingly, riboflavin/UVA combined with amphotericin B can enhance inhibitory effects on *A. fumigatus, C. albicans*, and *Fusarium*. Riboflavin/UV-A enhanced surface diffusion of amphotericin B and reduced vertical diffusion into the agar. This treatment strategy can be used to treat fungal infection keratitis [109]. Several studies have investigated the use of riboflavin to treat infectious keratitis. Clinical trials have shown that collagen cross-linking (CXL) with UV light-activated riboflavin has a positive role in the treatment of bacterial or fungal keratitis [110–113]. Conversely, no benefits of the CXL to treat infectious keratitis have been reported [114–117]. Based on current evidence, further investigations are needed to clarify the efficacy of CXL-UVA riboflavin for treatment of infectious keratitis. Moreover, riboflavin can also be used for virus inactivation [18]. Riboflavin and UV light reduced the infectious titer of severe acute respiratory distress syndrome coronavirus-2 [118,119], middle east respiratory syndrome coronavirus [120], dengue viruses [121], and ebolavirus [122] below the limit of detection. In summary, exogenous riboflavin plays a positive role in infectious diseases through combination with other drugs or therapies. Riboflavin’s use as a synergist and photosensitizer are summarized in Table 2.

**Table 2.** Riboflavin’s use as a synergist and photosensitizer

Usage	Wavelength of light	Target organisms	Reference
Synergist	-	MRSA	[101,102]
	-	hepatitis C virus	[100]
Photosensitizer	450 nm	*S. aureus*	[103]
	355 nm	*E. coli* and *P. aeruginosa*	[104]
	365 nm	*S. aureus; P. aeruginosa*; MRAS; *Staphylococcus epidermidis; A. fumigatus; C. albicans* and *Fusarium*	[104,105,108,109]
	<400 nm	SARS-CoV-2; MERS-CoV; dengue viruses; ebolavirus	[118–122]

### Exogenous riboflavin and immune responses

As an antioxidant, exogenous riboflavin has a vital effect on the treatment and prevention of infectious diseases directly or indirectly by regulating the immune response and redox state. The role of exogenous riboflavin in immune responses is summarized in Figure 2c. On the one hand, exogenous riboflavin facilitates the ability of immune cells to protect the host from infection. For instance, riboflavin regulates the accumulation, infiltration, migration, and development of macrophages and PMNs [98,123,124]. Under some conditions, pathogens are disseminated through immune cells (such as macrophages and PMNs) [125]. It reduced the level of HMGB1 released by macrophages [126], although the underlying mechanism is unknown [99]. On the other hand, exogenous riboflavin decreases cytokine/chemokine levels (including those of TNF-α, IL-1β, MCP-1, and IL-6) and increases the levels of IL-12p70 and IL-10 [127–131]. Riboflavin also regulates the level of heat shock proteins, including Hsp25 and Hsp72 [97] (Figure 2c). Heat shock proteins can protect against cellular injury and death under harmful environmental conditions, such as infection [97,132,133]. TNF-α is a well-known pro-inflammatory cytokine [133] that participates in inflammatory cell activation and recruitment [128]. TNF-α and INF-γ are partially responsible for LPS-induced *iNOS* mRNA expression [128]. Furthermore, TNF-α and INF-γ induced inflammatory cell death during SARS-CoV-2 infection, which may contribute to cytokine storm activation [127]. TNF-α is one of the reasons for the destruction of the immune defense during infection [129]. IL-10 primarily limits excessive inflammatory responses by inhibiting pro-inflammatory mediators, including TNF-α, IL-1β, IL-6, and GM-CSF [130]. It also maintains tissue homeostasis and innate immunity to control infection [133]. Thus, TNF, ILs, and IFN play vital roles in the occurrence and development of infection. However, further studies are needed to determine the effects of exogenous riboflavin on infections disease; its detailed effects on the immune system have yet to be elucidated.

## Conclusion

In this review, anti-infectious roles of the endogenous riboflavin biosynthesis pathway, FMN riboswitch, and exogenous riboflavin are summarized. The data suggest that the riboflavin biosynthesis pathway may be a promising target for the development of novel classes of antibiotics and antifungals. In bacteria, the FMN riboswitch is the main target of existing riboflavin biosynthesis inhibitors, such as RoF, AF, Ribocil and 5FDQD. In addition, enzymes, such as LS, RS, riboflavin kinase, and FAD synthase, can also be targets for antibiotics [30,34]. However, few studies have developed inhibitors of the riboflavin biosynthesis pathway in fungi. Further studies are also needed to clarify whether enzyme inhibitors of riboflavin biosynthesis will be useful as a novel class of antifungal agents. In addition, MAIT cells recognize bacteria and fungi in an MR1-dependent manner, which requires the riboflavin synthesis pathway, while viruses depend on cytokines (Figure 2b). How MAIT cells respond to bacteria/fungi treated with inhibitors of the riboflavin biosynthesis pathway will require further investigation. Notably, if the inhibitors inhibit the production of pyrimidine adducts, MR1 cannot present antigen to MAIT cells and trigger an immune response *in vivo*, which may cause the effect of these inhibitors *in vivo* to be worse than that *in vitro*.

Furthermore, during infection, exogenous riboflavin inhibits the mRNA expression of LPS and reduces the overproduction of NO (Figure 2b), as well as regulates innate immune cells and cytokines (Figure 2c). However, whether exogenous riboflavin influences the *de novo* synthesis of riboflavin needs to be further explored. It also remains to be determined whether MAIT cells can respond to exogenous riboflavin or whether exogenous riboflavin can be presented by MR1 and compete with endogenous riboflavin/riboflavin precursors. Further understanding of these mechanisms will shed light on the role of endogenous and exogenous riboflavin in infection. In summary, further investigation of the endogenous riboflavin biosynthesis pathway and exogenous riboflavin will help lay the foundation for the development of new antimicrobials.

## Data Availability

Data sharing is not applicable to this article as no new date were created or analyzed in this study.

## References

[1] Furuya EY, Lowy FD. Antimicrobial-resistant bacteria in the community setting. Nat Rev Microbiol. 2006 4 (1):36–45. PMID: 16357859. 16357859 10.1038/nrmicro1325

[2] Xiao YH, Giske CG, Wei ZQ, et al. Epidemiology and characteristics of antimicrobial resistance in China. Drug Resist Updat. 2011 ;14(4–5):236–250. PMID: 21807550. 21807550 10.1016/j.drup.2011.07.001

[3] Fisher MC, Hawkins NJ, Sanglard D, et al. Worldwide emergence of resistance to antifungal drugs challenges human health and food security. Science. 2018 ;360(6390):739–742. PMID: 29773744. 29773744 10.1126/science.aap7999

[4] Perlin DS, Rautemaa-Richardson R, Alastruey-Izquierdo A. The global problem of antifungal resistance: prevalence, mechanisms, and management. Lancet Infect Dis. 2017 Dec;17(12):e383–e392. PMID: 28774698. 28774698 10.1016/S1473-3099(17)30316-X

[5] Devi U, Locarnini S. Hepatitis B antivirals and resistance. Curr Opin Virol. 2013 ;3(5):495–500. PMID: 24016777. 24016777 10.1016/j.coviro.2013.08.006

[6] Lampejo T. Influenza and antiviral resistance: an overview. Eur J Clin Microbiol Infect Dis. 2020;39(7):1201–1208. PMID: 32056049. 32056049 10.1007/s10096-020-03840-9 PMC7223162

[7] McKeegan KS, Borges-Walmsley MI, Walmsley AR. Microbial and viral drug resistance mechanisms. Trends Microbiol. 2002;10(10Suppl):S8–14. PMID: 12377562 12377562 10.1016/s0966-842x(02)02429-0

[8] Perlin DS, Rautemaa-Richardson R, Alastruey-Izquierdo A. The global problem of antifungal resistance. prevalence, mechanisms, and management. Lancet Infect Dis. 2017;17(12):e383–e392. PMID: 28774698 28774698 10.1016/S1473-3099(17)30316-X

[9] Waglechner N, Wright GD. Antibiotic resistance: it’s bad, but why isn’t it worse? BMC Biol. 2017;15(1):84. PMID: 28915805 28915805 10.1186/s12915-017-0423-1 PMC5603022

[10] Berman J, Krysan DJ. Drug resistance and tolerance in fungi. Nat Rev Microbiol. 2020;18(6):319–331. PMID: 32047294 32047294 10.1038/s41579-019-0322-2 PMC7231573

[11] Fuller AT. Is p-aminobenzenesulphonamide the active agent of prontosil therapy? Lancet. 1937;229(5917):194–198.

[12] Guzzo MB, Nguyen HT, Pham TH, et al. Methylfolate trap promotes bacterial thymineless death by sulfa drugs. PLoS Pathog. 2016;12(10):e1005949. PMID: 27760199 27760199 10.1371/journal.ppat.1005949 PMC5070874

[13] Bertacine Dias MV, Santos JC, Libreros-Zúñiga GA, et al. Folate biosynthesis pathway: mechanisms and insights into drug design for infectious diseases. Future Med Chem. 2018;10(8):935–959. PMID: 29629843 29629843 10.4155/fmc-2017-0168

[14] Parente-Rocha JA, Bailão AM, Amaral AC, et al. Antifungal resistance, metabolic routes as drug targets, and new antifungal agents: an overview about endemic dimorphic fungi. Mediators Inflamm. 2017;2017:1-16. PMID: 28694566. 10.1155/2017/9870679 PMC5485324 28694566

[15] Pedrolli D, Langer S, Hobl B, et al. The ribB FMN riboswitch from Escherichia coli operates at the transcriptional and translational level and regulates riboflavin biosynthesis. FEBS. 2015;282(16):3230–3242. PMID: 25661987 10.1111/febs.13226 25661987

[16] Kundu B, Sarkar D, Ray N, et al. Understanding the riboflavin biosynthesis pathway for the development of antimicrobial agents. Med Res Rev. 2019;39(4):1338–1371. PMID: 30927319 30927319 10.1002/med.21576

[17] Pinto JT, Zempleni J. Riboflavin. Adv Nutr. 2016;7(5):973–975. PMID: 27633112 27633112 10.3945/an.116.012716 PMC5015041

[18] Thakur K, Tomar SK, Singh AK, et al. Riboflavin and health: a review of recent human research. Crit Rev Food Sci Nutr. 2017;57(17):3650–3660.PMID: 27029320 27029320 10.1080/10408398.2016.1145104

[19] Flieger M, Bandouchova H, Cerny J, et al. Vitamin B2 as a virulence factor in Pseudogymnoascus destructans skin infection. Sci Rep. 2016;6:33200. PMID: 27620349 27620349 10.1038/srep33200 PMC5020413

[20] Toyosawa T, Suzuki M, Kodama K, et al. Effects of intravenous infusion of highly purified vitamin B2 on lipopolysaccharide-induced shock and bacterial infection in mice. Eur J Pharmacol. 2004;492(2–3):273–280. PMID: 15178375 15178375 10.1016/j.ejphar.2004.04.004

[21] Mazur-Bialy AI, Kolaczkowska E, Plytycz B. Modulation of zymosan-induced peritonitis by riboflavin co-injection, pre-injection or post-injection in male Swiss mice. Life Sci. 2012;91(25–26):1351–1357. PMID: 23123448 23123448 10.1016/j.lfs.2012.10.016

[22] Fischer M, Bacher A. Biosynthesis of flavocoenzymes. Nat Prod Rep. 2005;22(3):324–350. PMID: 25635378 16010344 10.1039/b210142b

[23] Bacher A, Eberhardt S, Fischer M, et al. Biosynthesis of vitamin b2 (riboflavin). Annu Rev Nutr. 2000;20:153–167. PMID: 10940330 10940330 10.1146/annurev.nutr.20.1.153

[24] Förstermann U, Sessa WC. Nitric oxide synthases: regulation and function. Eur Heart. 2012;33(7):829–37, 837a-837d. PMID: 21890489 10.1093/eurheartj/ehr304 PMC3345541 21890489

[25] Liu S, Hu W, Wang Z, et al. Production of riboflavin and related cofactors by biotechnological processes. Microb Cell Fact. 2020;19(1):31. PMID: 32054466 32054466 10.1186/s12934-020-01302-7 PMC7017516

[26] Cecchini G, Perl M, Lipsick J, et al. Transport and binding of riboflavin by Bacillus subtilis. J Biol Chem. 1979 Aug 10;254(15):7295–7301. PMID: 110806. 110806

[27] Schneider C, Mack M. A second riboflavin import system is present in flavinogenic Streptomyces davaonensis and supports roseoflavin biosynthesis. Mol Microbiol. 2021 Apr 7. 10.1111/mmi.14726. PMID: 33829573. 33829573

[28] Vitreschak AG, Rodionov DA, Mironov AA, et al. Regulation of riboflavin biosynthesis and transport genes in bacteria by transcriptional and translational attenuation. Nucleic Acids Res. 2002 Jul 15;30(14):3141–3151. PMID: 12136096. 12136096 10.1093/nar/gkf433 PMC135753

[29] Gutiérrez-Preciado A, Torres AG, Merino E, et al. Extensive Identification of Bacterial Riboflavin Transporters and Their Distribution across Bacterial Species. PLoS One. 2015 May 4;10(5):e0126124. PMID: 25938806. 25938806 10.1371/journal.pone.0126124 PMC4418817

[30] Abbas CA, Sibirny AA. Genetic control of biosynthesis and transport of riboflavin and flavin nucleotides and construction of robust biotechnological producers. Microbiol Mol Biol Rev. 2011;75(2):321–360. PMID: 21646432 21646432 10.1128/MMBR.00030-10 PMC3122625

[31] Perl M, Kearney EB, Singer TP. Transport of riboflavin into yeast cells. J Biol Chem. 1976 Jun 10;251(11):3221–3228. PMID: 6447. 6447

[32] Reihl P, Stolz J. The monocarboxylate transporter homolog Mch5p catalyzes riboflavin (vitamin B2) uptake in Saccharomyces cerevisiae. J Biol Chem. 2005 Dec 2; Epub 2005 Oct 4. 280(48):39809–39817. PMID: 16204239. 16204239 10.1074/jbc.M505002200

[33] Barile M, Giancaspero TA, Leone P, et al. Riboflavin transport and metabolism in humans. J Inherit Metab Dis. 2016 Jul;39(4):545–557; Epub 2016 Jun 6. PMID: 27271694. 27271694 10.1007/s10545-016-9950-0

[34] Krajewski SS, Ignatov D, Johansson J. Two are better than one: dual targeting of riboswitches by metabolite analogs. Cell Chem Biol. 2017;24(5):535–537. PMID: 28525764 28525764 10.1016/j.chembiol.2017.05.004

[35] Blount KF, Breaker RR. Riboswitches as antibacterial drug targets. Nat Biotechnol. 2006;24(12):1558–1564. PMID: 17160062 17160062 10.1038/nbt1268

[36] Panchal V, Brenk R. Riboswitches as drug targets for antibiotics. Antibiotics. 2021;10(1):45. PMID: 33466288 33466288 10.3390/antibiotics10010045 PMC7824784

[37] Howe JA, Wang H, Fischmann TO, et al. Selective small-molecule inhibition of an RNA structural element. Nature. 2015;526(7575):672–677. PMID: 26416753 26416753 10.1038/nature15542

[38] Balibar CJ, Villafania A, Barbieri CM, et al. Validation and development of an Escherichia coli riboflavin pathway phenotypic screen hit as a small-molecule ligand of the flavin mononucleotide riboswitch. Methods Mol Biol. 2018;1787:19–40. PMID: 29736707 29736707 10.1007/978-1-4939-7847-2_2

[39] Motika SE, Ulrich RJ, Geddes EJ, et al. Gram-negative antibiotic active through inhibition of an essential riboswitch. J Am Chem Soc. 2020;142(24):10856–10862. PMID: 32432858 32432858 10.1021/jacs.0c04427 PMC7405991

[40] Wang H, Mann PA, L X, et al. Dual-targeting small-molecule inhibitors of the Staphylococcus aureus FMN riboswitch disrupt riboflavin homeostasis in an infectious setting. Cell Chem Biol. 2017;24(5):576–588.e6. 28434876 10.1016/j.chembiol.2017.03.014

[41] Jankowitsch F, Schwarz J, Rückert C, et al. Genome sequence of the bacterium Streptomyces davawensis JCM 4913 and heterologous production of the unique antibiotic roseoflavin. Genome sequence of the bacterium Streptomyces davawensis JCM 4913 and heterologous production of the unique antibiotic roseoflavin. J Bacteriol. 2012;194(24):6818–6827. PMID: 23043000 23043000 10.1128/JB.01592-12 PMC3510588

[42] Lee ER, Blount KF, Breaker RR. Roseoflavin is a natural antibacterial compound that binds to FMN riboswitches and regulates gene expression. RNA Biol. 2009;6(2):187–194. PMID: 19246992 19246992 10.4161/rna.6.2.7727 PMC5340298

[43] Serganov A, Huang L, Patel DJ. Coenzyme recognition and gene regulation by a flavin mononucleotide riboswitch. Nature. 2009;458(7235):233–237. PMID: 19169240 19169240 10.1038/nature07642 PMC3726715

[44] Mansjö M, Johansson J. The riboflavin analog roseoflavin targets an FMN-riboswitch and blocks Listeria monocytogenes growth, but also stimulates virulence gene-expression and infection. RNA Biol. 2011;8(4):674–680. PMID: 21593602 21593602 10.4161/rna.8.4.15586 PMC3225981

[45] Matern A, Pedrolli D, Großhennig S, et al. Uptake and metabolism of antibiotics roseoflavin and 8-Demethyl-8-Aminoriboflavin in riboflavin-auxotrophic Listeria monocytogenes. J Bacteriol. 2016;198(23):3233–3243. PMID: 27672192 27672192 10.1128/JB.00388-16 PMC5105903

[46] Pedrolli DB, Mack M. Bacterial flavin mononucleotide riboswitches as targets for flavin analogs. Methods Mol Biol. 2014;1103:165–176. PMID: 24318894 24318894 10.1007/978-1-62703-730-3_13

[47] Ott E, Stolz J, Lehmann M, et al. RFN riboswitch of Bacillus subtilis is a target for the antibiotic roseoflavin produced by Streptomyces davawensis. RNA Biol. 2009;6(3):276–280. PMID: 19333008 19333008 10.4161/rna.6.3.8342

[48] Grill S, Busenbender S, Pfeiffer M, et al. The bifunctional flavokinase/flavin adenine dinucleotide synthetase from Streptomyces davawensis produces inactive flavin cofactors and is not involved in resistance to the antibiotic roseoflavin. J Bacteriol. 2008;190(5):1546–1553. PMID: 18156273 18156273 10.1128/JB.01586-07 PMC2258686

[49] Pedrolli DB, Matern A, Wang J, et al. A highly specialized flavin mononucleotide riboswitch responds differently to similar ligands and confers roseoflavin resistance to Streptomyces davawensis. Nucleic Acids Res. 2012;40(17):8662–8673. PMID: 22740651 22740651 10.1093/nar/gks616 PMC3458559

[50] Langer S, Hashimoto M, Hobl B, et al. Flavoproteins are potential targets for the antibiotic roseoflavin in Escherichia coli. J Bacteriol. 2013;195(18):4037–4045. PMID: 23836860 23836860 10.1128/JB.00646-13 PMC3754745

[51] Pedrolli DB, Nakanishi S, Barile M, et al. The antibiotics roseoflavin and 8-demethyl-8-amino-riboflavin from Streptomyces davawensis are metabolized by human flavokinase and human FAD synthetase. Biochem Pharmacol. 2011;82(12):1853–1859. PMID: 21924249 21924249 10.1016/j.bcp.2011.08.029

[52] Blount KF, Megyola C, Plummer M, et al. Novel riboswitch-binding flavin analog that protects mice against Clostridium difficile infection without inhibiting cecal flora. Antimicrob Agents Chemother. 2015;59(9):5736–5746. PMID: 26169403 26169403 10.1128/AAC.01282-15 PMC4538501

[53] Long Q, Ji L, Wang H, et al. Riboflavin biosynthetic and regulatory factors as potential novel anti-infective drug targets. Chem Biol Drug Des. 2010;75(4):339–347. PMID: 20148904 20148904 10.1111/j.1747-0285.2010.00946.x

[54] Bonomi HR, Marchesini MI, Klinke S, et al. An atypical riboflavin pathway is essential for Brucella abortus virulence. PLoS One. 2010;5(2):e9435. PMID: 20195542 20195542 10.1371/journal.pone.0009435 PMC2828483

[55] Morgunova E, Saller S, Haase I, et al. Lumazine synthase from Candida albicans as an anti-fungal target enzyme: structural and biochemical basis for drug design. J Biol Chem. 2007;282(23):17231–17241. PMID: 17446177 17446177 10.1074/jbc.M701724200

[56] Kumar P, Singh M, Karthikeyan S. Crystal structure analysis of icosahedral lumazine synthase from Salmonella typhimurium, an antibacterial drug target. Acta Crystallogr D Biol Crystallogr. 2011;67(Pt2):131–139. PMID: 21245535 21245535 10.1107/S0907444910053370

[57] Chen J, Illarionov B, Bacher A, et al. A high-throughput screen utilizing the fluorescence of riboflavin for identification of lumazine synthase inhibitors. Anal Biochem. 2005;338(1):124–130. PMID: 15707942 15707942 10.1016/j.ab.2004.11.033

[58] Meir Z, Osherov N. Vitamin biosynthesis as an antifungal target. J Fungi. 2018;4(2):72. PMID: 29914189 10.3390/jof4020072 PMC6023522 29914189

[59] Zhao Y, Bacher A, Illarionov B, et al. Discovery and development of the covalent hydrates of trifluoromethylated pyrazoles as riboflavin synthase inhibitors with antibiotic activity against Mycobacterium tuberculosis. J Org Chem. 2009;74(15):5297–5303. PMID: 19545132 19545132 10.1021/jo900768c

[60] Serer MI, Carrica MDC, Trappe J, et al. A high-throughput screening for inhibitors of riboflavin synthase identifies novel antimicrobial compounds to treat brucellosis. FEBS J. 2019;286(13):2522–2535. PMID: 30927485 30927485 10.1111/febs.14829

[61] Cushman M, Jin G, Sambaiah T, et al. Design, synthesis, and biochemical evaluation of 1,5,6,7-tetrahydro-6,7-dioxo-9-D-ribitylaminolumazines bearing alkyl phosphate substituents as inhibitors of lumazine synthase and riboflavin synthase. J Org Chem. 2005;70(20):8162–8170. PMID: 16277343 16277343 10.1021/jo051332v PMC2548293

[62] Islam Z, Kumar A, Singh S, et al. Structural basis for competitive inhibition of 3,4-dihydroxy-2-butanone-4-phosphate synthase from Vibrio cholerae. J Biol Chem. 2015;290(18):11293–11308. PMID: 25792735 25792735 10.1074/jbc.M114.611830 PMC4416836

[63] Jin L, Zhou H, Zhao S, et al. Cloning and characterization of a new antibacterial target, 3,4-dihydroxy-2-butanone-4-phosphate synthase. Acta Microbiologica Sinica. 2012;52(11):1415–1420. PMID: 23383514. 23383514

[64] Li J, Hua Z, Miao L, et al. The crystal structure and biochemical properties of DHBPS from Streptococcus pneumoniae, a potential anti-infective target for Gram-positive bacteria. Protein Expr Purif. 2013;91(2):161–168. PMID: 23954596 23954596 10.1016/j.pep.2013.07.007

[65] Sebastián M, Velázquez-Campoy A, The MM. RFK catalytic cycle of the pathogen Streptococcus pneumoniae shows species-specific features in prokaryotic FMN synthesis. J Enzyme Inhib Med Chem. 2018;33(1):842–849. PMID: 29693467 29693467 10.1080/14756366.2018.1461857 PMC6010069

[66] Lans I, Anoz-Carbonell E, Palacio-Rodríguez K, et al. In silico discovery and biological validation of ligands of FAD synthase, a promising new antimicrobial target. PLoS Computational Biology. 2020;16(8):e1007898. PMID: 32797038. 10.1371/journal.pcbi.1007898 PMC7449411 32797038

[67] Dietl AM, Meir Z, Shadkchan Y, et al. Riboflavin and pantothenic acid biosynthesis are crucial for iron homeostasis and virulence in the pathogenic mold Aspergillus fumigatus. Virulence. 2018;9(1):1036–1049. PMID: 30052132 30052132 10.1080/21505594.2018.1482181 PMC6068542

[68] Demuyser L, Palmans I, Vandecruys P, et al. Molecular elucidation of riboflavin production and regulation in Candida albicans, toward a novel antifungal drug target. mSphere. 2020;5(4):e00714–20. PMID: 32759338 32759338 10.1128/mSphere.00714-20 PMC7407072

[69] Becker JM, Kauffman SJ, Hauser M, et al. Pathway analysis of Candida albicans survival and virulence determinants in a murine infection model. Proc Natl Acad Sci USA. 2010;107(51):22044–22049. PMID: 21135205 21135205 10.1073/pnas.1009845107 PMC3009777

[70] Garfoot AL, Zemska O, Rappleye CA. Histoplasma capsulatum depends on de novo vitamin biosynthesis for intraphagosomal proliferation. Infect Immun. 2014 Jan;82(1):393–404; Epub 2013 Nov 4. PMID: 24191299. 24191299 10.1128/IAI.00824-13 PMC3911860

[71] de Castro PA, Chiaratto J, Morais ER, et al. The putative flavin carrier family FlcA-C is important for Aspergillus fumigatus virulence. Virulence. 2017;8(6):797–809. PMID: 27652896 27652896 10.1080/21505594.2016.1239010 PMC5626198

[72] Ussher JE, Klenerman P, Willberg CB. Mucosal-associated invariant T-cells: new players in anti-bacterial immunity. Front Immunol. 2014;5:450. PMID: 25339949 25339949 10.3389/fimmu.2014.00450 PMC4189401

[73] Eckle SB, Corbett AJ, Keller AN, et al. Recognition of Vitamin B precursors and byproducts by mucosal associated invariant T cells. J Biol Chem. 2015;290(51):30204–30211.PMID: 26468291 26468291 10.1074/jbc.R115.685990 PMC4683245

[74] Franciszkiewicz K, Salou M, Legoux F, et al. MHC class I-related molecule, MR1, and mucosal-associated invariant T cells. Immunol Rev. 2016;272(1):120–138. PMID: 27319347 27319347 10.1111/imr.12423

[75] Keller AN, Corbett AJ, Wubben JM, et al. MAIT cells and MR1-antigen recognition. Curr Opin Immunol. 2017;46:66–74. PMID: 28494326 28494326 10.1016/j.coi.2017.04.002

[76] Constantinides MG, Link VM, Tamoutounour S, et al. MAIT cells are imprinted by the microbiota in early life and promote tissue repair. Science. 2019;366(6464):eaax6624. PMID: 31649166 31649166 10.1126/science.aax6624 PMC7603427

[77] Chen Z, Wang H, D’Souza C, et al. Mucosal-associated invariant T-cell activation and accumulation after in vivo infection depends on microbial riboflavin synthesis and co-stimulatory signals. Mucosal Immunol. 2017;10(1):58–68. PMID: 27143301 27143301 10.1038/mi.2016.39

[78] Wang H, D’Souza C, Lim XY et al. MAIT cells protect against pulmonary Legionella longbeachae infection. Nature Communucations.2018;9(1):3350. 10.1038/s41467-018-05202-8 PMC6105587 30135490

[79] Kurioka A, Ussher JE, Cosgrove C, et al. MAIT cells are licensed through granzyme exchange to kill bacterially sensitized targets. Mucosal Immunol. 2015;8(2):429–440. PMID: 25269706 25269706 10.1038/mi.2014.81 PMC4288950

[80] Sakala IG, Kjer-Nielsen L, Eickhoff CS, et al. Functional heterogeneity and antimycobacterial effects of mouse mucosal-associated invariant T Cells specific for riboflavin metabolites. J Immunol. 2015;195(2):587–601. PMID: 26063000 26063000 10.4049/jimmunol.1402545 PMC4490942

[81] Bucsan AN, Rout N, Foreman TW, et al. Mucosal-activated invariant T cells do not exhibit significant lung recruitment and proliferation profiles in macaques in response to infection with Mycobacterium tuberculosis CDC1551. Tuberculosis. 2019;116S:S11–S18. PMID: 31072689 31072689 10.1016/j.tube.2019.04.006 PMC7050191

[82] Napier RJ, Adams EJ, Gold MC, et al. The role of mucosal associated invariant T Cells in antimicrobial immunity. Front Immunol. 2015;6:344. PMID: 26217338 26217338 10.3389/fimmu.2015.00344 PMC4492155

[83] Le Bourhis L, Martin E, Péguillet I, et al. Antimicrobial activity of mucosal-associated invariant T cells. Nat Immunol. 2010;11(8):701–708. PMID: 20581831 20581831 10.1038/ni.1890

[84] Jahreis S, Böttcher S, Hartung S, et al. Human MAIT cells are rapidly activated by Aspergillus spp. in an APC-dependent manner. Eur J Immunol. 2018;48(10):1698–1706. PMID: 30059139 30059139 10.1002/eji.201747312

[85] Böttcher S, Hartung S, Meyer F, et al. Human mucosal-associated invariant T cells respond to Mucorales species in a MR1-dependent manner. Med Mycol. 2020:myaa103. DOI:10.1093/mmy/myaa103. PMID: 33336238. 33336238

[86] Dias J, Leeansyah E, Sandberg JK. Multiple layers of heterogeneity and subset diversity in human MAIT cell responses to distinct microorganisms and to innate cytokines. Proc Natl Acad Sci USA. 2017;114(27):E5434–E5443. PMID: 28630305 28630305 10.1073/pnas.1705759114 PMC5502643

[87] van Wilgenburg B, Loh L, Chen Z, et al. MAIT cells contribute to protection against lethal influenza infection in vivo. Nat Commun. 2018;9(1):4706. PMID: 30413689 30413689 10.1038/s41467-018-07207-9 PMC6226485

[88] van Wilgenburg B, Scherwitzl I, Hutchinson EC, et al. MAIT cells are activated during human viral infections. Nat Commun. 2016;7:11653. PMID: 27337592 27337592 10.1038/ncomms11653 PMC4931007

[89] Ussher JE, Willberg CB, Klenerman P. MAIT cells and viruses. Immunol Cell Biol. 2018;96(6):630–641. PMID: 29350807 29350807 10.1111/imcb.12008 PMC6055725

[90] Huang W, He W, Shi X, et al. Mucosal-associated invariant T-cells are severely reduced and exhausted in humans with chronic HBV infection. J Viral Hepat. 2020;27(11):1096–1107. PMID: 32510704 32510704 10.1111/jvh.13341

[91] Leeansyah E, Ganesh A, Quigley MF. Activation, exhaustion, and persistent decline of the antimicrobial MR1-restricted MAIT-cell population in chronic HIV-1 infection. Blood. 2013;121(7):1124–1135. PMID: 23243281 23243281 10.1182/blood-2012-07-445429 PMC3575756

[92] Leeansyah E, Svärd J, Dias J. Arming of MAIT cell cytolytic antimicrobial activity is induced by IL-7 and defective in HIV-1 infection. PLoS Pathog. 2015;11(8):e1005072. PMID: 26295709 26295709 10.1371/journal.ppat.1005072 PMC4546682

[93] Tang X, Zhang S, Sustained PQ. IFN-I stimulation impairs MAIT cell responses to bacteria by inducing IL-10 during chronic HIV-1 infection. Sci Adv. 2020;6(8):eaaz0374. PMID: 32128419 32128419 10.1126/sciadv.aaz0374 PMC7030930

[94] Shih CK, Chen CM, Chen CY, et al. Riboflavin protects mice against liposaccharide-induced shock through expression of heat shock protein 25. Food Chem Toxicol. 2010;48(7):1913–1918. PMID: 20430062 20430062 10.1016/j.fct.2010.04.033

[95] Toyosawa T, Suzuki M, Kodama K, et al. Potentiation by amino acid of the therapeutic effect of highly purified vitamin B2 in mice with lipopolysaccharide-induced shock. Eur J Pharmacol. 2004;493(1–3):177–182. PMID: 15189780 15189780 10.1016/j.ejphar.2004.04.019

[96] Kodama K, Suzuki M, Toyosawa T, et al. Inhibitory mechanisms of highly purified vitamin B2 on the productions of proinflammatory cytokine and NO in endotoxin-induced shock in mice. Life Sci. 2005;78(2):134–139. PMID: 16112685 16112685 10.1016/j.lfs.2005.04.037

[97] Mazur-Bialy AI, Pochec E, Plytycz B. Immunomodulatory effect of riboflavin deficiency and enrichment-reversible pathological response versus silencing of inflammatory activation. J Physiol Pharmacol. 2015;66(6):793–802. PMID: 26769828 26769828

[98] Mazur-Bialy AI, Majka A, Wojtas L, et al. Strain-specific effects of riboflavin supplementation on zymosan-induced peritonitis in C57BL/6J, BALB/c and CBA mice. Life Sci. 2011;88(5–6):265–271. PMID: 21115019 21115019 10.1016/j.lfs.2010.11.016

[99] Mazur-Bialy AI, Pocheć E. HMGB1 inhibition during zymosan-induced inflammation: the potential therapeutic action of riboflavin. Arch Immunol Ther Exp. 2016;64(2):171–176. PMID: 26445809 10.1007/s00005-015-0366-6 PMC4805693 26445809

[100] Saito H, Ebinuma H, Tada S, et al. Enhancing effect of the liver extract and flavin adenin dinucleotide mixture on anti-viral efficacy of interferon in patients with chronic hepatitis C. Keio J Med. 1996;45(1):48–53. PMID: 8882468 8882468 10.2302/kjm.45.48

[101] Shahzad S, Ashraf MA, Sajid M, et al. Evaluation of synergistic antimicrobial effect of vitamins (A, B1, B2, B6, B12, C, D, E and K) with antibiotics against resistant bacterial strains. Journal of Global Antimicrobial Resistance. 2018 (13):231–236.PMID: 29408383. 10.1016/j.jgar.2018.01.005 29408383

[102] Mal P, Dutta K, Bandyopadhyay D, et al. Azithromycin in combination with riboflavin decreases the severity of Staphylococcus aureus infection induced septic arthritis by modulating the production of free radicals and endogenous cytokines. Inflamm Res. 2013;62(3):259–273.PMID: 23229721 23229721 10.1007/s00011-012-0574-z

[103] Banerjee S, Ghosh D, Vishakha K, et al. Photodynamic antimicrobial chemotherapy (PACT) using riboflavin inhibits the mono and dual species biofilm produced by antibiotic resistant Staphylococcus aureus and Escherichia coli. Photodiagnosis Photodyn Ther. 2020;32:102002. PMID: 32916327 32916327 10.1016/j.pdpdt.2020.102002

[104] Rivas Aiello MB, Ghilini F, Martínez Porcel JE, et al. Riboflavin-mediated photooxidation of gold nanoparticles and its effect on the inactivation of bacteria. Langmuir. 2020;36(28):8272–8281. PMID: 32569473 32569473 10.1021/acs.langmuir.0c01473

[105] Schrier A, Greebel G, Attia H, et al. In vitro antimicrobial efficacy of riboflavin and ultraviolet light on Staphylococcus aureus, methicillin-resistant Staphylococcus aureus, and Pseudomonas aeruginosa. J Refract Surg. 2009;25(9):S799–802. PMID: 19772254 19772254 10.3928/1081597X-20090813-07

[106] Hiraku Y, Ito K, Hirakawa K, et al. Photosensitized DNA damage and its protection via a novel mechanism. Photochem Photobiol. 2007;83(1):205–212. PMID: 16965181 16965181 10.1562/2006-03-09-IR-840

[107] Baier J, Maisch T, Maier M, et al. Singlet oxygen generation by UVA light exposure of endogenous photosensitizers. Biophys J. 2006;91(4):1452–1459. PMID: 16751234 16751234 10.1529/biophysj.106.082388 PMC1518628

[108] Bäckman A, Makdoumi K, Mortensen J, et al. The efficiency of cross-linking methods in eradication of bacteria is influenced by the riboflavin concentration and the irradiation time of ultraviolet light. Acta Ophthalmol. 2014;92(7).PMID: 25493311 10.1111/aos.12301 25493311

[109] Sauer A, Letscher-Bru V, Speeg-Schatz C, et al. In vitro efficacy of antifungal treatment using riboflavin/UV-A (365 nm) combination and amphotericin B. Invest Ophthalmol Vis Sci. 2010;51(8):3950–3953. PMID: 20335618 20335618 10.1167/iovs.09-4013

[110] Davis SA, Bovelle R, Han G, et al. Corneal collagen cross-linking for bacterial infectious keratitis. Cochrane Database Syst Rev. 2020;6(6):CD013001. PMID: 32557558 32557558 10.1002/14651858.CD013001.pub2 PMC7389372

[111] Li Z, Jhanji V, Tao X, et al. Riboflavin/ultravoilet light-mediated crosslinking for fungal keratitis. Br J Ophthalmol. 2013;97(5):669–671. PMID: 23355529 23355529 10.1136/bjophthalmol-2012-302518

[112] Said DG, Elalfy MS, Gatzioufas Z, et al. Collagen cross-linking with photoactivated riboflavin (PACK-CXL) for the treatment of advanced infectious keratitis with corneal melting. Ophthalmology. 2014;121(7):1377–1382. PMID: 24576886 24576886 10.1016/j.ophtha.2014.01.011

[113] Bilgihan K, Kalkanci A, Ozdemir HB, et al. Evaluation of antifungal efficacy of 0.1% and 0.25% riboflavin with UVA: a comparative in vitro study. Curr Eye Res. 2016;41(8):1050–1056. PMID: 26644282 26644282 10.3109/02713683.2015.1088956

[114] Prajna NV, Radhakrishnan N, Lalitha P, et al. Cross-linking-assisted infection reduction: a randomized clinical trial evaluating the effect of adjuvant cross-linking on outcomes in fungal keratitis. Ophthalmology. 2020;127(2):159–166. PMID: 31619359 31619359 10.1016/j.ophtha.2019.08.029 PMC6982573

[115] Kashiwabuchi RT, Carvalho FR, Khan YA, et al. Assessment of fungal viability after long-wave ultraviolet light irradiation combined with riboflavin administration. Graefes Arch Clin Exp Ophthalmol. 2013;251(2):521–527. PMID: 23180236 23180236 10.1007/s00417-012-2209-z

[116] Chan TC, Lau TW, Lee JW, et al. Corneal collagen cross-linking for infectious keratitis: an update of clinical studies. Acta Ophthalmol. 2015;93(8):689–696. PMID: 25990098 25990098 10.1111/aos.12754

[117] Arboleda A, Miller D, Cabot F, et al. Assessment of rose bengal versus riboflavin photodynamic therapy for inhibition of fungal keratitis isolates. Am J Ophthalmol. 2014;158(1):64–70.e2. PMID: 24792103 24792103 10.1016/j.ajo.2014.04.007 PMC4075940

[118] Ragan I, Hartson L, Pidcoke H, et al. Pathogen reduction of SARS-CoV-2 virus in plasma and whole blood using riboflavin and UV light. PLoS One. 2020;15(5):e0233947.PMID: 32470046 32470046 10.1371/journal.pone.0233947 PMC7259667

[119] Keil SD, Ragan I, Yonemura S, et al. Inactivation of severe acute respiratory syndrome coronavirus 2 in plasma and platelet products using a riboflavin and ultraviolet light-based photochemical treatment. Vox Sang. 2020;115(6):495–501. PMID: 32311760 32311760 10.1111/vox.12937 PMC7264728

[120] Keil SD, Bowen R, Marschner S. Inactivation of middle east respiratory syndrome coronavirus (MERS-CoV) in plasma products using a riboflavin-based and ultraviolet light-based photochemical treatment. Transfusion. 2016;56(12)PMID: 27805261 10.1111/trf.13860 PMC7169765 27805261

[121] Faddy HM, Fryk JJ, Watterson D, et al. Riboflavin and ultraviolet light. impact on dengue virus infectivity. Vox Sang. 2016;111(3):235–241. PMID: 27281512 27281512 10.1111/vox.12414

[122] Cap AP, Pidcoke HF, Keil SD, et al. Treatment of blood with a pathogen reduction technology using ultraviolet light and riboflavin inactivates Ebola virus in vitro. Transfusion. 2016;56:S6–15. Suppl 1 (Suppl1). PMID: 27001363. 27001363 10.1111/trf.13393 PMC5943045

[123] Dey S, Bishayi B. Riboflavin along with antibiotics balances reactive oxygen species and inflammatory cytokines and controls Staphylococcus aureus infection by boosting murine macrophage function and regulates inflammation. J Inflamm. 2016;13(36). DOI:10.1186/s12950-016-0145-0.PMID: 27932936 PMC5126841 27932936

[124] Tran JQ, Muench MO, Heitman JW, et al. Pathogen reduction with riboflavin and ultraviolet light induces a quasi-apoptotic state in blood leukocytes. Transfusion. 2019;59(11):3501–3510. PMID: 31599981 31599981 10.1111/trf.15516 PMC7391079

[125] Van Dyck K, Viela F, Mathelié-Guinlet M, et al. Adhesion of Staphylococcus aureus to Candida albicans During Co-Infection Promotes Bacterial Dissemination Through the Host Immune Response. Front Cell Infect Microbiol. 2021 Feb;2(10):624839. PMID: 33604309. 33604309 10.3389/fcimb.2020.624839 PMC7884861

[126] Andersson U, Tracey KJ. HMGB1 is a therapeutic target for sterile inflammation and infection. Annu Rev Immunol. 2011;29:139–162. PMID: 21219181 21219181 10.1146/annurev-immunol-030409-101323 PMC4536551

[127] Karki R, Sharma BR, Tuladhar S, et al. Synergism of TNF-α and IFN-γ triggers inflammatory cell death, tissue damage, and mortality in SARS-CoV-2 infection and cytokine shock syndromes. Cell. 2021;184(1):149–168.e17. PMID: 33278357 33278357 10.1016/j.cell.2020.11.025 PMC7674074

[128] Salkowski CA, Detore G, McNally R, et al. Regulation of inducible nitric oxide synthase messenger RNA expression and nitric oxide production by lipopolysaccharide in vivo: the roles of macrophages, endogenous IFN-gamma, and TNF receptor-1-mediated signaling. J Immunol. 1997;158(2):905–912. PMID: 8993010 8993010

[129] Popescu M, Cabrera-Martinez B, Winslow GMTNF-Α. contributes to lymphoid tissue disorganization and germinal center B cell suppression during intracellular bacterial infection. J Immunol. 2019;203(9):2415–2424. PMID: 31570507 31570507 10.4049/jimmunol.1900484 PMC6810925

[130] de Waal Malefyt R, Abrams J, Bennett B, et al. Interleukin 10(IL-10) inhibits cytokine synthesis by human monocytes: an autoregulatory role of IL-10 produced by monocytes. J Exp Med. 1991;174(5).PMID: 1940799 10.1084/jem.174.5.1209 PMC2119001 1940799

[131] De Maio A. Heat shock proteins: facts, thoughts, and dreams. Shock. 1999;11(1):1–12. PMID: 9921710. 10.1097/00024382-199901000-00001 9921710

[132] Parameswaran N, Patial S. Tumor necrosis factor-α signaling in macrophages. Crit Rev Eukaryot Gene Expr. 2010;20(2):87–103. PMID: 21133840 21133840 10.1615/critreveukargeneexpr.v20.i2.10 PMC3066460

[133] Ouyang W, O’Garra A. IL-10 family cytokines IL-10 and IL-22: from basic science to clinical translation. immunity. 2019;50(4):871–891. PMID: 30995504 30995504 10.1016/j.immuni.2019.03.020

